# Brain function abnormalities and inflammation in HIV-positive men who have sex with men with depressive disorders

**DOI:** 10.3389/fpsyt.2024.1438085

**Published:** 2025-01-20

**Authors:** Yang Zhang, Yihui He, Yuan Fang, Miaotian Cai, Guangqiang Sun, Rui Wang, Jiaxin Zhen, Yulin Zhang, Zhen Li, Yundong Ma, Tong Zhang

**Affiliations:** ^1^ Center for Infectious Disease, Beijing Youan Hospital, Capital Medical University, Beijing, China; ^2^ Beijing Institute for Sexually Transmitted Disease Control, Beijing, China; ^3^ Postgraduate Union Training Base of Jinzhou Medical University, PLA Rocket Force Characteristic Medical Center, Beijing, China; ^4^ Department of Neurology, PLA Rocket Force Characteristic Medical Center, Beijing, China; ^5^ Department of Respiratory and Critical Care Medicine, Beijing Youan Hospital, Capital Medical University, Beijing, China; ^6^ Beijing Key Laboratory of Mental Disorders, National Clinical Research Center for Mental Disorders and National Center for Mental Disorders, Beijing Anding Hospital, Capital Medical University, Beijing, China; ^7^ Advanced Innovation Center for Human Brain Protection, Capital Medical University, Beijing, China; ^8^ Beijing Key Laboratory of HIV/AIDS Research, Beijing, China

**Keywords:** human immunodeficiency virus, depressive disorders, resting-state functional magnetic resonance imaging, peripheral immunity, inflammation

## Abstract

**Background:**

Depressive disorders are highly prevalent among people with HIV (PWH) and are related to aberrant inflammation and immune responses. However, there is currently a lack of investigation into the neurological, inflammatory, endocrine, and immune aspects of HIV-associated depressive disorders (HADD).

**Methods:**

The study involved 33 HIV-positive men who have sex with men with depressive disorders (HADD group) and 47 without neuropsychiatric disorders (HIV control group). Participants underwent resting-state functional magnetic resonance imaging (rs-fMRI) scans and assessments of peripheral blood. Peripheral blood cytokines, plasma concentrations of hormone and neurotrophic factors, and immune cell levels were determined using liquid chip, enzyme-linked immunosorbent assay, and flow cytometry, respectively. The correlation of imaging alterations with clinical variables and peripheral blood indicators was assessed.

**Results:**

Compared to the HIV control group, the HADD group exhibited a higher fractional amplitude of low-frequency fluctuations in the left superior parietal gyrus, lower regional homogeneity in the left precentral gyrus, and reduced voxel-wise functional connectivity for the seed region in the right precentral gyrus with clusters in the right cuneus, etc. Furthermore, the HADD group had higher levels of interferon-gamma, a higher frequency of non-classical monocytes, and higher expression levels of perforin and CD38 on specific cells. These imaging results were significantly correlated with peripheral blood indicators and clinical variables.

**Conclusion:**

This rs-fMRI study provides considerable evidence for abnormal intrinsic brain activity in people with HADD. Furthermore, our data also indicate the detrimental effects of depression-related inflammation on PWH. Therefore, it is imperative to increase attention to HADD and implement effective preventive interventions accordingly.

## 1 Introduction

HIV-associated depressive disorders (HADD) are highly prevalent in the era of potent antiretroviral therapy, even when patients are virally suppressed (1). The presence of depressive disorders negatively affects medication adherence, disease progression, and mortality in people with HIV (PWH), placing a serious burden on patients, their families, and society (2). Several factors associated with HIV/acquired immunodeficiency syndrome (AIDS) status contribute to the high prevalence of depressive disorders, including infectious-immunological, psychosocial, and external factors (3–5). Substantial evidence suggests that HIV reservoirs in the central nervous system (CNS) may cause brain injury through chronic inflammation (6). Chronic inflammation and immune activation significantly contribute to non-AIDS-related neuropsychiatric adverse events (including HADD) in PWH (7, 8). Nonetheless, the pathogenesis of HADD is highly complex and the relationship between the neurological, inflammation, and immune systems of HADD is unclear. Investigations of brain function may help clarify the impact of HADD on the brain.

Magnetic resonance imaging (MRI) is the most frequently employed method in depressive disorder studies, and it has provided invaluable insights into the neuropathology of HIV (9). Previous studies have illustrated that PWH present brain activation abnormalities and gray matter atrophy compared to healthy controls (10, 11). Nevertheless, current studies on HADD by MRI are lacking. Firstly, more attention has been paid to the neuropsychiatric conditions in PWH. However, depressive disorders are extremely underdiagnosed in HIV/AIDS largely due to the lack of professional evaluation by highly-trained psychiatrists. Some clinicians prefer to use scales and questionnaires rather than specialized diagnostic tools for neuropsychiatric disorders. Secondly, in the field of HIV research, studies mainly focus on patients with HIV infection as well as those without infection. In particular, there have been numerous studies on HIV-associated neurocognitive disorders using MRI in this area. However, there is limited research on HIV-associated neuropsychiatric conditions such as depressive disorders in imaging. Thus, empirical data on brain imaging alterations in people with HADD is scarce.

To investigate changes in brain function among people with HADD, we examined the resting-state functional MRI (rs-fMRI) in a cohort of men who have sex with men (MSM) with HADD, comparing them to a well-matched group of HIV-positive MSM without neuropsychiatric disorders. Additionally, given the association between depression, inflammation, neurotrophins, endocrine, and immunity (12–15), we aimed to explore the effects of these factors on brain imaging as well as depressive disorders among PWH. Finally, correlation analyses were conducted to explore the relationships between imaging alterations, clinical data, inflammation-related markers, neurotrophic factors, endocrine indicators, and immune variables. The results of this investigation can provide potential theoretical foundations and data support for future studies on neuronal imaging and inflammation related to HADD.

## 2 Materials and methods

### 2.1 Participants

This cross-sectional study obtained approval from the Institutional Ethics Committee of Beijing Youan Hospital, Capital Medical University (2023/057). Prior to signing a written informed consent form, all participants were informed of the entire process and potential risks. The inclusion criteria for this study were as follows: (1) virologically suppressed HIV-infected individuals; (2) Chinese MSM; (3) aged at least 18 years; (4) right-handed; (5) not taking antidepressants; and (6) capable of signing an informed consent form. The exclusion criteria were: (1) individuals with current or previous opportunistic CNS infections; (2) individuals with a history of neurological disorders such as epilepsy, multiple sclerosis, Parkinson’s disease, or dementia; (3) individuals with MRI contraindications or claustrophobia; (4) previously experienced head injury with loss of consciousness for more than 30 minutes; and (5) substance abuse. Eventually, 106 participants were enrolled in our research project between May 2022 and November 2022.

We selected participants with HADD (defined as the HADD group) and those without neuropsychiatric disorders (defined as the HIV control group) for subsequent analysis. Sixteen participants with other types of neuropsychiatric disorders were excluded from the study. All participants underwent clinical, MRI, and peripheral blood assessments on the same day.

### 2.2 Clinical assessments

#### 2.2.1 Diagnosis of neuropsychiatric disorders

Psychiatric diagnoses were determined by a psychiatrist following the diagnostic criteria in the *Diagnostic and Statistical Manual of Mental Disorders*, 5th edition (16).

#### 2.2.2 Neurocognitive, mood, and sleep assessments

Neurocognitive function assessment was conducted using the Montreal Cognitive Assessment (MoCA) (17). Anxiety and depression levels of all participants were evaluated using the Self-Anxiety Scale (SAS) and the Self-Depression Scale (SDS), respectively (18, 19). Psychological health status was evaluated using the Symptom Checklist 90 (SCL-90) (20, 21). Sleep quality was assessed by the Pittsburgh Sleep Quality Index (PSQI) (22).

#### 2.2.3 Other assessments

Childhood maltreatment history was evaluated utilizing the Childhood Trauma Questionnaire (CTQ) (23). The assessment of alcohol craving included the administration of the Alcohol Urge Questionnaire (AUQ) and the Visual Analogue Scale (VAS) (24, 25).

### 2.3 MRI data acquisition

All imaging data were obtained using a 1.5 T MRI scanner (Philips, Amsterdam, The Netherlands) at the Second Hospital of Beijing. Foam cushions were utilized to restrain head movement. All participants were instructed to lie down, relax, close their eyes, and not think about anything specific to avoid falling asleep.

Whole-brain resting-state functional MRI data were acquired using a gradient echo planar imaging sequence. The acquisition parameters were as follows: repetition time/echo time (TR/TE) = 4019.8/30 ms, slices = 40, matrix = 64 × 62, flip angle = 90^°^, slice thickness = 3.5 mm, no gap, volumes = 102, and scanning time = 6 min 52 s. The structural images were used for the registration process of functional images, with the following acquisition parameters: shortest TR/TE = 8.3/3.9 ms, matrix = 256 × 227, field of view = 256 mm × 256 mm, flip angle = 12^°^, slice thickness = 1 mm, and slice number = 384.

### 2.4 Image preprocessing

Image preprocessing and statistical analyses were conducted using Matlab R2023a (The MathWorks, Natick, MA, USA). Initially, the raw data were inspected for anatomical abnormalities and scanner artifacts. All image data obtained in digital imaging and communications in medicine format were converted to neuroimaging informatics technology initiative format for further processing and analysis.

The rs-fMRI data were preprocessed using Statistical Parametric Mapping 12 (SPM12, https://www.fil.ion.ucl.ac.uk/spm/software/spm12/) and the Rs-fMRI Data Analysis Toolkit (REST, http://www.restfmri.net) (26). The processing pipeline included the following steps: first, removal of initial volumes (n = 5), followed by slice timing correction and motion correction. Then, the functional images were co-registered with individual T1-weighted structural images and spatially normalized by the Montreal Neurological Institute brain template (27). The normalized functional images were resampled to an isotropic voxel size (3.0 × 3.0 × 3.0 mm), smoothed with a full-width half maxima (FWHM) Gaussian kernel (6 mm), and underwent linear drift removal. The Friston 24-parameter head motion model was applied to eliminate the impacts of head motion (28, 29). To further remove nuisance signals, regression was performed on the average white matter and cerebrospinal fluid signals. Finally, a temporal band-pass filter (0.01-0.08 Hz) was applied to eliminate drifts and physiological noise.

Nine participants were removed from further analysis due to excessive head motion (displacements > 2.0 mm or rotations > 2.0^°^).

### 2.5 Computation of brain rs-fMRI metrics

#### 2.5.1 Computation of amplitude of low-frequency fluctuations (ALFF)/fractional ALFF maps

The ALFF value of each voxel was obtained by computing the mean square root of the power spectrum within the frequency range (0.01-0.08 Hz). The ALFF maps of all participants were computed to measure spontaneous brain activity (30). The fALFF measures the ratio of the power spectrum within a specific frequency range to the power spectrum across the entire frequency range. In order to standardize the variation between participants, the ALFF/fALFF maps of each individual were normalized to the mean ALFF/fALFF map and used for between-group comparisons.

#### 2.5.2 Computation of regional homogeneity (ReHo) maps

The ReHo value for each voxel was computed using Kendall’s coefficient of concordance for that voxel and its 26 neighboring voxels (31). Subsequently, Gaussian smoothing with a FWHM of 6 mm was applied to ReHo maps to diminish residual differences and noise in gyral anatomy. To mitigate the overall impact of differences between subjects, we computed the mean ReHo for each subject for group comparison.

#### 2.5.3 Computation of seed-based whole-brain functional connectivity (FC) maps

Previous neuroimaging studies have indicated that depressive disorders are related to focal functional and structural abnormalities in various brain regions, including the precentral gyrus, dorsolateral prefrontal cortex, medial prefrontal cortex, insula, hippocampus, anterior cingulate cortex, posterior cingulate cortex, precuneus, and caudate nucleus (32, 33). For FC analysis, the average blood-oxygen level-dependent time series from twenty-two seed regions were calculated. These seed regions included the bilateral precentral gyrus, medial part of the superior frontal gyrus, dorsolateral part of the superior frontal gyrus, medial orbital part of the superior frontal gyrus, anterior cingulate and paracingulate gyri, median cingulate and paracingulate gyri, posterior cingulate gyrus, insula, hippocampus, amygdala, precuneus, and caudate nucleus. In addition, brain regions showing group differences in ALFF, fALFF, and ReHo analyses were also selected as seed regions for FC analysis. The detailed information of these seed regions is shown in **Supplementary Table 1**. These seed region masks were derived from the automated anatomical labeling atlas (34). Before conducting intergroup comparisons using these FC maps, Fisher *r*-to-*z* conversion was applied to elevate the normality of the FC maps.

### 2.6 Cytokine and chemokine assay: Luminex^®^ xMAP^®^ technology

Inflammation has been reported to be associated with depression as well as some brain functional alterations (35–37). We examined the plasma levels of 37 cytokines and chemokines in the participants. The cytokine and chemokine assays on plasma samples were conducted using the MILLIPLEX^®^ MAP Human Cytokine/Chemokine/Growth Factor Panel A MAGNETIC BEAD PANEL 96-Well Plate Assay (EMD Millipore, Billerica, MA, USA), which is based on the cutting-edge Luminex^®^ xMAP^®^ technology. All steps were conducted according to the manufacturer’s instructions. Detailed methods can be found in the **Supplementary Material**.

### 2.7 Assessment of hormones and neurotrophic factors

The correlations of endocrine hormones and neurotrophic factors were determined using enzyme-linked immunosorbent assay kits. We primarily analyzed seven hormones and neurotrophic factors. For detailed information on these analyses, please refer to **Supplementary Table 2**. All measurements were conducted according to the manufacturers’ protocols.

### 2.8 Mass cytometry and data analysis

This study used 23 custom antibodies to identify various immune cells. These antibodies were purchased preconjugated from Fluidigm (South San Francisco, USA). Detailed information about the antibodies and reporter isotopes is available in **Supplementary Table 3**. The cell labeling process followed established protocols (38). Please refer to the methods section of the **Supplementary Material** for detailed methods and operational procedures. Using a doublet-filtering approach, the raw data of each pre-processed sample was de-barcoded utilizing distinct mass-tagged barcodes. The.fcs files produced by various batches were standardized using the bead normalization technique. Subsequently, meticulous gating was performed using FlowJo software (version 10.9.0) to remove debris and dead cells. Lymphocytes and monocytes were then artificially gated for further analysis using the R language. The PhenoGraph clustering technique was used to separate the cells into many clusters based on the expression levels of surface markers. To reduce dimensionality and visualize the high-dimensional data, a visual dimensionality reduction approach called *t*-distributed stochastic neighbor embedding was used. The distribution of each cluster, the expression of markers, and differences between groups or sample types were analyzed using R software (version 4.2.3).

### 2.9 Statistical analysis

The statistical analysis was conducted using SPSS software (version 25.0; IBM Corp., Armonk, New York, USA). The significance threshold (α) was set at 0.05. The normality of the data was assessed using the Kolmogorov-Smirnov and Shapiro-Wilk tests. For normally distributed data, continuous variables were expressed as mean ± standard deviation; for non-normally distributed data, continuous variables were expressed as median and interquartile range. Based on the results of the normality test, a two-sample *t*-test or Mann-Whitney *U*-test was employed to compare continuous variables between groups. Categorical data were presented as proportions. Chi-square tests and Fisher’s exact tests were employed to compare categorical variables between groups. In correlation analysis, Pearson correlation analysis was performed for normally distributed data, and Spearman correlation analysis was performed for non-normally distributed data. An additional analysis was conducted to explore whether the observed group differences were attributable to the primary effects of depressive disorders, ART regimens, or the interaction between them.

In voxel-based comparisons, two sample *t*-tests were used to examine differences between the two groups in terms of ALFF, fALFF, ReHo, and FC. Age and years of education were used as covariates in the analysis process. The significance level was determined using a voxel threshold of P < 0.001 and a cluster threshold of P < 0.05 (corrected for multiple comparisons using false discovery rate (FDR), Gaussian random field, and AlphaSim correction). For further exploratory analyses, a threshold of *P* < 0.001 was applied (uncorrected for multiple comparisons). Imaging results were displayed using MRIcroGL software (https://www.nitrc.org/projects/mricrogl/). The imaging results were correlated with clinical data and peripheral blood metrics. Statistical results were graphed using GraphPad Prism software (version 9.5.1; GraphPad Software, San Diego, CA, USA).

## 3 Results

### 3.1 Demographic and clinical characteristics of participants

A total of 80 participants successfully completed the study, with 33 participants (41.25%) in the HADD group and 47 participants (58.75%) in the HIV control group. Most demographic variables were well-matched between the two groups. The demographics and clinical characteristics are comprehensively outlined in **Table 1**.

**Table 1:** Demographic and clinical characteristics of all participants.

Demographic and clinical data	HADD group (N = 33)	HIV control group (N = 47)	Statistic	*P* value
Age (years)	33.00 (26.50 - 40.00)	33.00 (29.00 - 38.00)	*Z* = -0.166	0.868^a^
Height (m)	1.74 ± 0.05	1.75 ± 0.06	*t* = -0.913	0.364^b^
Weight (kg)	68.24 ± 10.27	69.32 ± 9.47	*t* = -0.483	0.630^b^
BMI (kg/m²)	22.15 (20.37 - 24.05)	22.10 (20.68 - 23.89)	*Z* = -0.093	0.926^a^
Education (years)	16.00 (14.50 - 16.50)	16.00 (15.00 - 16.00)	*Z* = -0.010	0.992^a^
Period of diagnosed HIV infection				
CD4 at diagnosis (cells/μL)	353.32 ± 151.96	329.55 ± 208.75	*t* = 0.589	0.557^b^
CD8 at diagnosis (cells/μL)	962.93 (844.50 - 1305.00)	936.00 (741.00 - 1183.00)	*Z* = -1.173	0.241^a^
CD4/CD8 ratio at diagnosis	0.33 (0.26 - 0.47)	0.38 (0.15 - 0.45)	*Z* = -0.259	0.796^a^
VL at diagnosis (log10 copies/mL)	4.03 (3.80 - 4.90)	4.07 (3.56 - 4.72)	*Z* = -0.963	0.336^a^
Period of initial ART start				
CD4 at initiation of ART (cells/μL)	381.82 ± 177.36	326.8 ± 208.07	*t* = 1.236	0.220^b^
CD8 at initiation of ART (cells/μL)	1114.95 (745.00 - 1329.09)	936 (749.00 - 1209.82)	*Z* = -1.300	0.194^a^
CD4/CD8 ratio at initiation of ART	0.33 (0.25 - 0.51)	0.37 (0.15 - 0.45)	*Z* = -0.562	0.574^a^
VL at initiation of ART (log10 copies/mL)	3.93 (3.77 - 4.71)	4.08 (3.56 - 4.82)	*Z* = -0.112	0.910^a^
ART regimen at initiation (INSTI/Non-INSTI - based regimen)	6/27	10/37	*χ^2^* = 0.116	0.733^c^
Period of clinical and MRI assessment				
Current CD4 (cells/μL)	589.00 (450.00 - 808.32)	559.00 (385.00 - 750.00)	*Z* = -0.821	0.412^a^
Current CD8 (cells/μL)	1003.47 (573.00 - 1250.00)	854.00 (645.00 - 1028.00)	*Z* = -0.582	0.561^a^
Current CD4/CD8 ratio	0.68 (0.41 - 0.90)	0.72 (0.43 - 0.90)	*Z* = -0.171	0.864^a^
Current virus not detectable (yes/no)	33/0	47/0	NA	NA
Current ART regimen (INSTI/Non-INSTI - based regimen)	20/13	34/13	*χ^2^* = 1.217	0.270^c^
Duration between diagnosis and initiation of ART (months)	0.60 (0.35 - 5.15)	0.50 (0.40 - 2.30)	*Z* = -0.383	0.702^a^
Duration of ART (months)	61.90 (20.20 - 97.25)	63.80 (43.10 - 88.00)	*Z* = -0.186	0.853^a^
Duration of HIV diagnosis (months)	78.50 (26.40 - 103.80)	70.80 (44.50 - 92.70)	*Z* = -0.034	0.973^a^
SAS	41.00 (33.00 - 44.00)	31.00 (24.00 - 34.00)	*Z* = -4.566	<0.001^a^
SDS	42.00 (34.50 - 49.50)	30.00 (26.00 - 36.00)	*Z* = -4.192	<0.001^a^
PSQI	7.00 (4.00 - 10.00)	4.00 (3.00 - 7.00)	*Z* = -2.913	0.004^a^
CTQ	61.00 (52.00 - 64.00)	57.00 (52.00 - 61.00)	*Z* = -1.645	0.100^a^
SCL-90	166.00 (132.50 - 215.50)	110.00 (99.00 - 133.00)	*Z* = -4.507	<0.001^a^
AUQ	9.00 (8.00 - 18.00)	11.00 (8.00 - 14.00)	*Z* = -0.610	0.542^a^
VAS	2.00 (1.00 - 4.00)	2.00 (1.00 - 4.00)	*Z* = -0.639	0.523^a^
MoCA	27.00 (25.50 - 28.00)	27.00 (25.00 - 28.00)	*Z* = -0.396	0.692^a^

The continuous data were expressed as mean ± standard deviation or median interquartile range and the categorical data were expressed as numbers. Two-sample *t*-tests were used for continuous data with a normal distribution, while Mann-Whitney *U*-tests were used for continuous data that did not obey a normal distribution. Chi-square and Fisher’s exact tests were used to compare categorical variables. aMann-Whitney *U*-test; btwo-sample *t*-test; cchi-square test. HADD, HIV-associated depressive disorders; HIV control, HIV-infected individuals without neuropsychiatric disorders; NA, not available; BMI, body mass index; CD4, CD4+ T cell count; CD8, CD8+ T cell count; VL, viral load; ART, antiretroviral therapy; INSTI, integrase strand transfer inhibitor; MRI, magnetic resonance imaging; SAS, self-rating anxiety scale; SDS, self-rating depression scale; SCL-90, symptom checklist 90; PSQI, Pittsburgh sleep quality index; CTQ, childhood trauma questionnaire; AUQ, alcohol urge questionnaire; VAS, visual analogue scale for alcohol craving; MoCA, Montreal cognitive assessment.

In comparison to the HIV control group, the HADD group exhibited significantly higher SAS scores (*P* < 0.001), SDS scores (*P* < 0.001), PSQI scores (*P* = 0.004), and SCL-90 scores (*P* < 0.001) (see **Table 1** and **Supplementary Table 4** for details). There were no differences between the two groups in CTQ, AUQ, VAS for alcohol craving, MoCA, or medication status (**Table 1**).

### 3.2 Comparison of brain rs-fMRI metrics

#### 3.2.1 ALFF/fALFF

**Figure 1:**
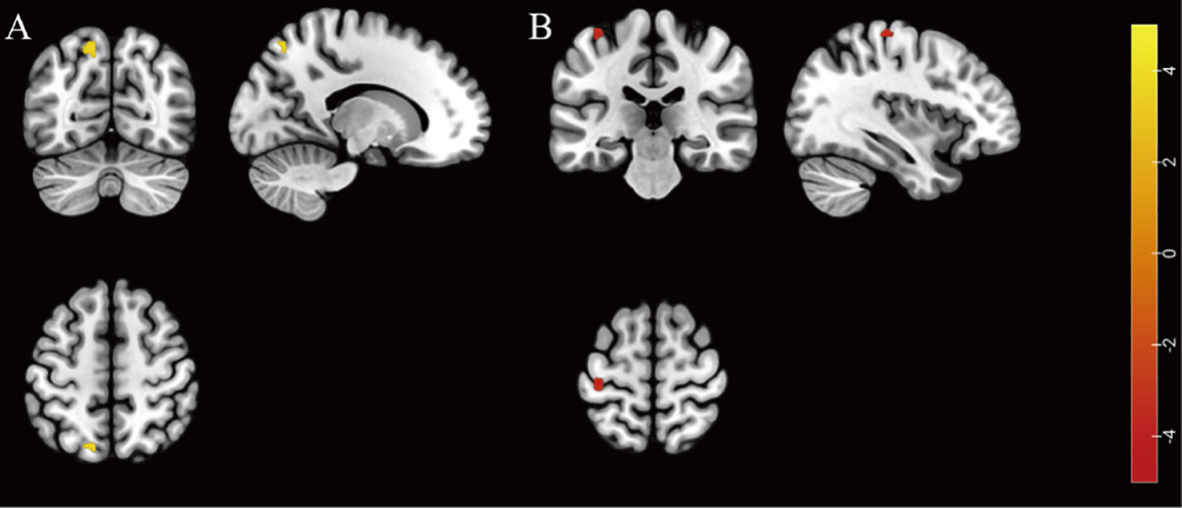
Individuals with HIV-associated depressive disorders exhibit abnormal functional activity in their brains. **(A)** The brain region with higher fALFF was located at the left superior parietal gyrus in the HADD group compared to the HIV control group (FDR correction, voxel-level *P* < 0.001, cluster-level *P* < 0.05). **(B)** The brain region with lower ReHo was located at the left precentral gyrus in the HADD group compared to the HIV control group (voxel-level uncorrected *P* < 0.001). fALFF, fractional amplitude of low-frequency fluctuation; ReHo, regional homogeneity; HADD, HIV-associated depressive disorders; HIV control, HIV-infected individuals without neuropsychiatric disorders; FDR, false discovery rate. The color bars indicate T-statistics (red/yellow).

The HADD group exhibited higher fALFF in the left superior parietal gyrus compared to the HIV control group (FDR correction, voxel-level *P* < 0.001, cluster-level *P* < 0.05; see **Figure 1** and **Supplementary Table 5** for details). In this final result, the largest cluster size consists of less than 10 continuous voxels, and there was no difference in the ALFF comparison between the two groups.

#### 3.2.2 ReHo

The HADD group exhibited lower ReHo in the left precentral gyrus compared to the HIV control group (voxel-level uncorrected *P* < 0.001; see **Figure 1** and **Supplementary Table 6** for details).

#### 3.2.3 FC

Compared to the HIV control group, the HADD group displayed decreased voxel-wise FC for the seed region in the right precentral gyrus with clusters in the right cuneus, the right medial orbital part of the superior frontal gyrus with clusters in the left inferior parietal, but supramarginal and angular gyri, and the right insula with clusters in the right calcarine fissure and surrounding cortex. (AlphaSim correction, voxel-level *P* < 0.001, cluster-level *P* < 0.05; **Table 2**).

**Table 2:** Functional connectivity differences between the HADD and HIV control groups.

Seed region number	Contrast/Seed regions	Connected regions	Peak MNI coordinates			T value	Cluster Size
			X	Y	Z		
	**HADD < HIV control**						
2	R precentral gyrus	R cuneus	15	-87	39	5.1858	33^a^
		L middle occipital gyrus	-33	-87	15	4.1676	25
		L middle frontal gyrus	-27	51	15	4.7906	23
		L inferior parietal, but supramarginal and angular gyri	-33	-39	45	4.2157	10
8	R medial orbital part of the superior frontal gyrus	L inferior parietal, but supramarginal and angular gyri	-36	-54	51	4.4441	21^a^
10	R insula	R calcarine fissure and surrounding cortex	21	-63	6	4.6126	27^b^
		R lingual gyrus	24	-54	0	3.9103	12
		R postcentral gyrus	54	-21	57	3.6817	10
14	R posterior cingulate gyrus	L inferior temporal gyrus	-48	-51	-21	4.2577	12

Coordinates (X, Y, Z) refer to the peak MNI coordinates of brain regions with peak intensity. ^a^Corrected for multiple comparisons (GRF correction, voxel-level *P* < 0.001, cluster-level *P* < 0.05, two-tailed); ^b^Corrected for multiple comparisons (AlphaSim correction, voxel-level *P* < 0.001, cluster-level *P* < 0.05). GRF, gaussian random field; HADD, HIV-associated depressive disorders; HIV control, HIV-infected individuals without neuropsychiatric disorders; FC, functional connectivity; MNI, Montreal Neurological Institute; L, left; R, right.

### 3.3 Peripheral blood indicators

**Figure 2:**
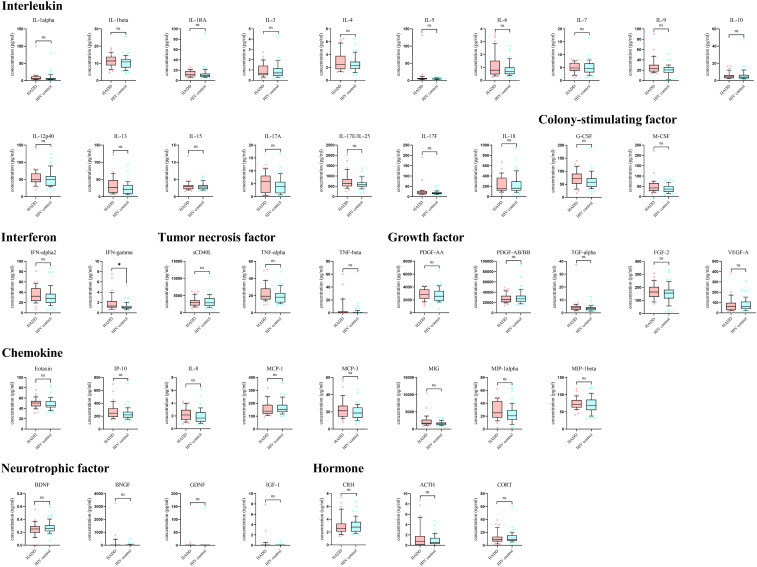
Differences in inflammatory, endocrine, and neurotrophic indicators between the HADD and HIV control groups. HADD, HIV-associated depressive disorders; HIV control, HIV-infected individuals without neuropsychiatric disorders; IL, interleukin; IL-1RA, interleukin-1 receptor antagonist; G/M-CSF, granulocyte/macrophage colony-stimulating factor; IFN, interferon; sCD40L, soluble CD40 ligand; TNF, tumor necrosis factor; PDGF, platelet-derived growth factor; TGF, transforming growth factor; FGF, fibroblast growth factor; VEGF, vascular endothelial growth factor; IP, interferon-inducible protein; MCP, monocyte chemotactic protein; MIG, monokine induced by gamma interferon; MIP, macrophage inflammatory protein; BDNF, brain-derived neurotrophic factor; BNGF, beta-nerve growth factor; GDNF, glial-derived neurotrophic factor; IGF-1, insulin-like growth factor-1; CRH, corticotropin-releasing hormone; ACTH, adrenocorticotropic hormone; CORT, cortisol; ns, non-significant. *P<0.05.

Higher plasma levels of interferon-gamma (IFN-γ) were found in the HADD group compared to the HIV control group (**Figure 2**).

### 3.4 Flow cytometry analysis results

**Figure 3:**
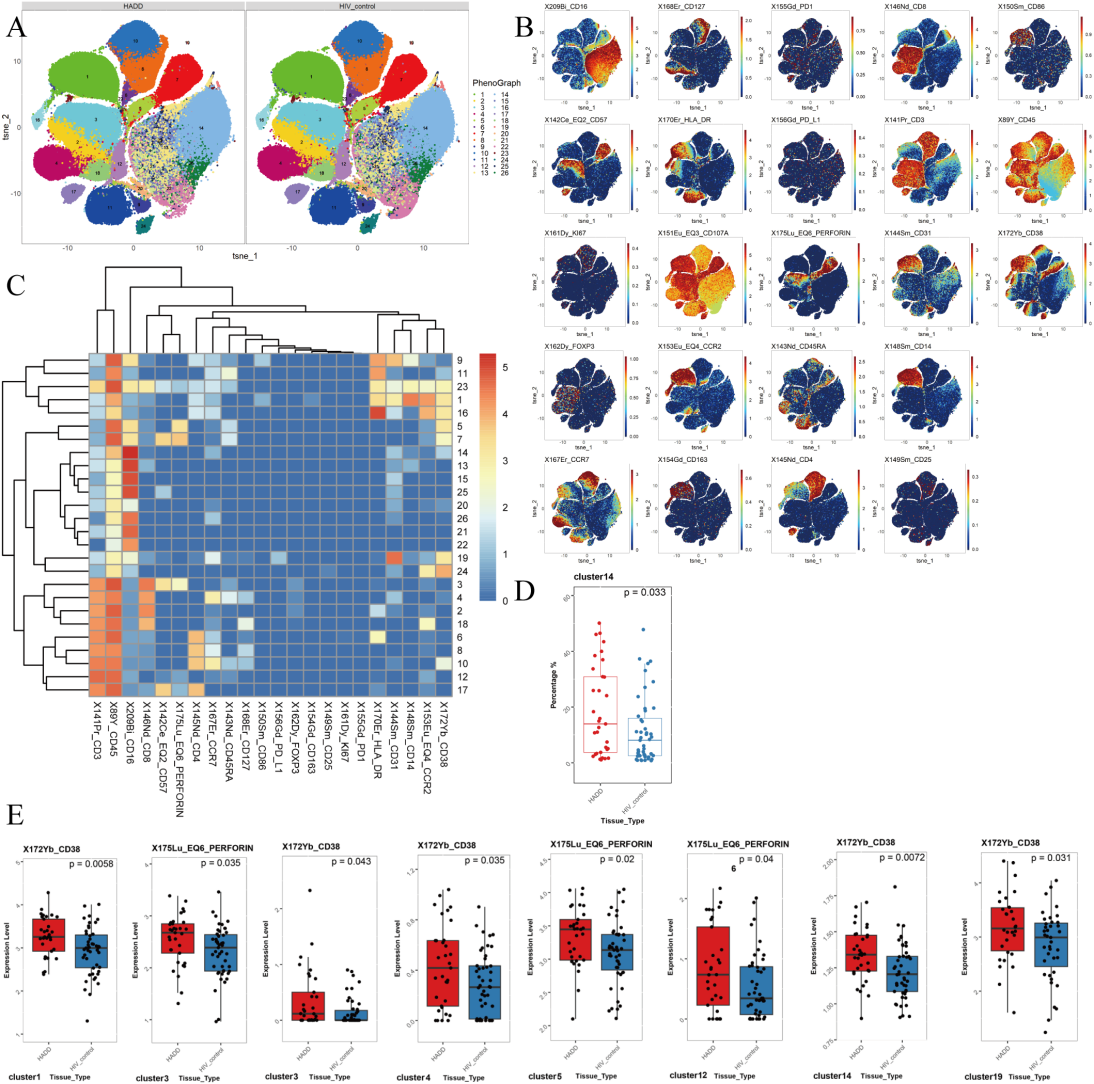
The results of flow cytometric analysis. **(A)** *t*-SNE plots of monocytes and CD3^+^ T cells in HADD and HIV control groups. **(B)** Expression distribution of selected markers across clusters. Dark red color: high expression; dark blue color: no expression. **(C)** Heatmap of marker expression for 26 monocytes and CD3^+^ T cell clusters. Among the 26 clusters of monocytes and CD3^+^ T cells, cluster 1 represented classical monocytes (CD14^+^ CD16^-^); cluster 2, 3, 18 represented CD8^+^ effector memory T cells (CD3^+^ CD4^-^ CD8^+^ CD45RA^-^ CCR7^-^); cluster 4, 23 represented CD8^+^ naïve T cells (CD3^+^ CD4^-^ CD8^+^ CD45RA^+^ CCR7^+^); cluster 5, 7, 11, 13, 14, 15, 19, 20, 21, 22, 24, 25, 26 represented nonclassical monocytes (CD14^-^ CD16^+^); cluster 17 represented CD4^+^ effector memory T cells (CD3^+^ CD4^+^ CD8^-^ CD45RA^-^ CCR7^-^); cluster 9 represented intermediate monocytes (CD14^+^ CD16^+^); cluster 10 represented CD4^+^ naïve T cells (CD3^+^ CD4^+^ CD8^-^ CD45RA^+^ CCR7^+^); cluster 12 represented double negative T cells (CD3^+^ CD4^-^ CD8^-^); cluster 6, 8, 16 represented CD4^+^ central memory T cells (CD3^+^ CD4^+^ CD8^-^ CD45RA^-^ CCR7^+^). **(D)** Different frequencies of cluster 14 (nonclassical monocytes) were observed between the HADD and HIV control groups. **(E)** Cluster showing significant differences in perforin and CD38 expression levels between the HADD and HIV control groups. Statistical significance was defined as a *P* value < 0.05. HADD, HIV-associated depressive disorders; HIV control, HIV-infected individuals without neuropsychiatric disorders; *t*-SNE, *t*-distributed stochastic neighbor embedding.

Compared to the HIV control group, we observed an elevated frequency of non-classical monocytes (NCM) in the HADD group. The results showed that the HADD group expressed greater levels of perforin (in cluster 3, which represents the CD8^+^ effector memory T cells (T_EM_); cluster 5, which represents the NCM; cluster 12, which represents the double-negative T cells (DNT)) and CD38 (in cluster 1, which represents the classical monocytes (CM); cluster 3; cluster 4, which represents the CD8^+^ naïve T cells (T_N_); cluster 14, which represents the NCM; cluster 19, which represents the NCM) compared to the HIV control group (**Figure 3**).

### 3.5 Analysis of the main effects and interactions between groups and ART regimens on findings

Depressive disorder showed significant main effects on the intergroup differences in most imaging, cytokine, and immunological indicators between the two groups. No main effects of ART regimens or interactions between groups and ART regimens were identified in the results (**Supplementary Table 7**).

### 3.6 Correlations of the imaging results with the clinical data and peripheral blood indicators

**Figure 4:**
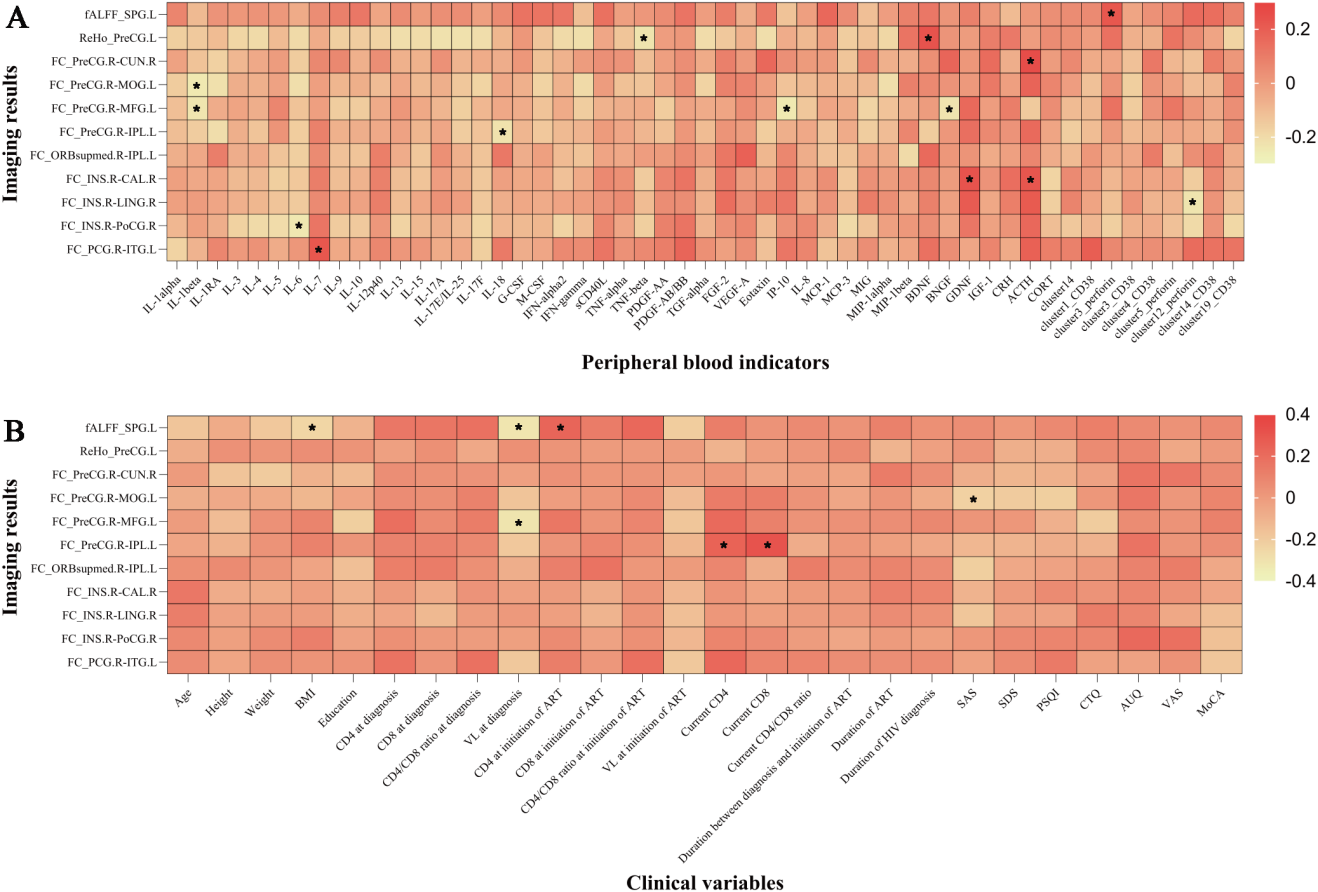
The imaging results were correlated with peripheral blood indicators and clinical variables. **(A)** Correlation between imaging results and peripheral blood indicators. **(B)** Correlation between imaging results and clinical variables. ^*^ *P* < 0.05. IL, interleukin; IL-1RA, interleukin-1 receptor antagonist; G/M-CSF, granulocyte/macrophage colony-stimulating factor; IFN, interferon; sCD40L, soluble CD40 ligand; TNF, tumor necrosis factor; PDGF, platelet-derived growth factor; TGF, transforming growth factor; FGF, fibroblast growth factor; VEGF, vascular endothelial growth factor; IP, interferon-inducible protein; MCP, monocyte chemotactic protein; MIG, monokine induced by gamma interferon; MIP, macrophage inflammatory protein; BDNF, brain-derived neurotrophic factor; BNGF, beta-nerve growth factor; GDNF, glial-derived neurotrophic factor; IGF-1, insulin-like growth factor-1; CRH, corticotropin-releasing hormone; ACTH, adrenocorticotropic hormone; CORT, cortisol; ALFF, fractional amplitude of low-frequency fluctuation; ReHo, regional homogeneity; FC, functional connectivity; SPG.L, left superior parietal gyrus; PreCG.L, left precentral gyrus; PreCG.R, right precentral gyrus; CUN.R, right cuneus; MOG.L, left middle occipital gyrus; MFG.L, left middle frontal gyrus; IPL.L, left inferior parietal, but supramarginal and angular gyri; ORBsupmed.R, right medial orbital part of the superior frontal gyrus; INS.R, right insula; CAL.R, right calcarine fissure and surrounding cortex; LING.R, right lingual gyrus; PoCG.R, right postcentral gyrus; PCG.R, right posterior cingulate gyrus; ITG.L, left inferior temporal gyrus; BMI, body mass index; CD4, CD4^+^ T cell count; CD8, CD8^+^ T cell count; VL, viral load; ART, antiretroviral therapy; INSTI, integrase strand transfer inhibitor; MRI, magnetic resonance imaging; SAS, self-rating anxiety scale; SDS, self-rating depression scale; PSQI, Pittsburgh sleep quality index; CTQ, childhood trauma questionnaire; AUQ, alcohol urge questionnaire; VAS, visual analogue scale for alcohol craving; MoCA, Montreal cognitive assessment. Cluster 14 represented nonclassical monocytes, cluster 1 represented nonclassical monocytes, cluster 3 represented CD8^+^ effector memory T cells, cluster 4 represented CD8^+^ naïve T cells, cluster 5 represented nonclassical monocytes, cluster 12 represented double negative T cells, and cluster 19 represented nonclassical monocytes.

In the analysis of all participants, there was a positive correlation between the brain-derived neurotrophic factor (BDNF) levels and ReHo values in the left precentral gyrus. The FC values (voxel-wise FC for the seed region in the right precentral gyrus with clusters in the left middle occipital gyrus) were negatively correlated with SAS scores, etc. (**Figure 4**).

## 4 Discussion

In this study, we conducted comparisons between the HADD and HIV control groups in terms of clinical, neuroimaging, neurotrophic factors, endocrinological indicators, and immunological markers. Compared to the HIV control group, we found that the HADD group exhibited higher fALFF in the left superior parietal gyrus, lower ReHo in the left precentral gyrus, and decreased voxel-wise FC for the seed region in the right precentral gyrus with clusters in the right cuneus, etc. Additionally, we observed higher levels of inflammation-related immune markers in the HADD group. These imaging changes were correlated with clinical variables and peripheral blood indicators. These results may provide evidence that depressive disorders are associated with brain function abnormalities and inflammation in HIV-infected MSM.

In brain functional imaging comparisons, we observed higher fALFF in the left superior parietal gyrus and lower ReHo in the left precentral gyrus in the HADD group compared to the HIV control group. The parietal gyrus is involved in the processing of sensory, emotional, and cognitive functions in the human brain (39). Relevant literature in neurology and psychiatry has reported that people with schizophrenia exhibit higher fALFF in the left superior parietal lobule compared to healthy control groups (40). We also discovered that the HADD group exhibited higher fALFF in the left superior frontal gyrus, indicating abnormal activity in this region in people with HADD. The precentral gyrus, located in the dorsal portion of the frontal lobe, is responsible for processing sensory input and motor output as well as planning, controlling, and executing voluntary movements (41, 42). Interestingly, previous studies have reported similar changes between people with major depressive disorder and healthy controls (43, 44), suggesting that abnormal ReHo in the precentral gyrus may serve as a prospective neuroimaging biomarker for depressive disorder (44). Therefore, our results suggest that people with HADD may exhibit abnormal brain activity in multiple brain regions compared to HIV controls.

According to the definition of seed regions of interest, we observed decreased voxel-wise FC in the HADD group for the seed region in the right insula with clusters in the right calcarine fissure and surrounding cortex, right postcentral gyrus, and right lingual gyrus. Previous research has also indicated similar results in the general population (45). These findings provide further proof that depression is closely associated with functional coordination deficits of the insula with the calcarine, postcentral gyrus, and lingual gyrus. It is suggested that this may serve as a relatively stable biomarker for depressive disorders. Thus, these findings indicate that individuals with HADD exhibit related abnormalities in brain network connections.

Current research on the mechanism of depression in PWH is relatively common, and it is now believed that HIV infection leads to dysfunction of the hypothalamic-pituitary-adrenal axis, causing sustained immune activation, which in turn leads to the occurrence of depression (37, 46). We found that levels of IFN-γ and the frequency of NCM were higher in the HADD group compared to the HIV control group. IFN-γ is a pro-inflammatory cytokine that plays a pivotal role in regulating immune and inflammatory events (47). This aligns with literature describing how HIV infection increases the release of tumor necrosis factor-α, IFN-γ, interleukin (IL)-1, and IL-6, resulting in reduced 5-hydroxytryptamine transmission and ultimately leading to depression in PWH (37). There are three subpopulations of monocytes in whole blood: classical monocytes (phagocytosis), intermediate monocytes (pro-inflammatory, phagocytosis), and NCM (patrolling, antiviral, pro-inflammatory) (48, 49). In some inflammatory diseases, NCM have been shown to exhibit pro-inflammatory features (50–52). The frequency of NCM is higher in the HADD group, and the levels of perforin and CD38 expression in NCM are also elevated, indicating increased inflammation levels in HADD patients. DNT cells are a unique antigen-specific regulatory T cell (53, 54), and the increased expression of perforin in the HADD group indicates enhanced cytotoxic function in these cells. Since DNT cells also play a role in promoting neuroinflammation (55), their active function in the HADD group may have implications for the promotion of inflammation. Overall, the results of these peripheral blood assessments suggest a higher level of inflammation in individuals with HADD.

In the correlation investigation, we discovered a positive correlation between the ReHo values of the left precentral gyrus and BDNF levels. BDNF, as the most abundant member of the neurotrophic factor family of growth factors in the CNS, plays key roles in neuronal survival and growth as well as synaptic plasticity (56). Previous studies have shown an etiological link between depression and BDNF (57–59). One of the most significant biological findings in depression disorders is the decline in peripheral (plasma or serum) BDNF levels (59). ReHo measures the consistency of time signals between a voxel and its surrounding voxels (31). Our study found that lower ReHo in the left precentral gyrus among individuals with HADD, which was related to lower levels of BDNF. The correlation between BDNF levels and the ReHo values of the left precentral gyrus seems to indicate a consistent correlation between imaging changes and neurotrophic factors.

This study has certain strengths. First, the combined use of multiple technologies and interdisciplinary methods allows for further investigation into the associations between clinical, imaging, endocrine, and immune results. Second, the comprehensive psychiatric disorders were diagnosed by expert psychiatrists using standard diagnostic criteria to ensure the accuracy of this study, avoiding potential biases that have arisen from using psychiatric screening measures such as the general health questionnaire in other clinical studies.

However, there are also limitations and shortcomings in this research. First, the cross-sectional design limited our capacity to study temporal relationships and draw causal inferences. Second, the sample of this study, primarily composed of HIV-positive MSM, limits the generalizability of the findings to other demographics. To improve applicability, future research should include more diverse populations, encompassing varied genders, sexual orientations, and socioeconomic backgrounds. Expanding the target population to other high-risk groups and the general population is also recommended to validate the robustness and broader relevance of the findings. Third, the relatively small sample size in this study may reduce statistical power. Future research should recruit larger and more diverse sample populations through multi-center cohort studies to ensure the findings are representative of broader HIV-positive populations. Fourth, the 1.5 T MRI scanner utilized in this study has inherent limitations in imaging resolution and signal-to-noise ratio. Compared to higher-field scanners, it may be less effective in capturing fine structural details and subtle signal variations. Future studies employing higher-field scanners could enhance resolution and signal-to-noise ratio, yielding more precise imaging data to validate our findings and provide deeper mechanistic insights for clinical applications.

In summary, this study has revealed that individuals with HADD display abnormalities in brain function and higher levels of chronic inflammation compared to PWH without neuropsychiatric disorders. Moreover, there is a clear correlation between imaging results, clinical data, and inflammation markers. These findings suggest that integrating imaging and immunological data in future research may deepen our understanding of the neurofunctional changes associated with HADD.

## Data Availability

The raw data supporting the conclusions of this article will be made available by the authors, without undue reservation.
