# Supplementary material for: Brain function abnormalities and inflammation in HIV-positive men who have sex with men with depressive disorders
Source: Front Psychiatry. 2025 Jan 20;15:1438085. doi: 10.3389/fpsyt.2024.1438085 (PMC11788281; doi:10.3389/fpsyt.2024.1438085)
Supplement: Supplementary file 1 [file DataSheet1.docx]

Supplementary Material

**Supplementary Data**

**Methods**

**Cytokine and chemokine assay: Luminex® xMAP® technology**

Peripheral blood samples from each subject were collected in the morning (8-10 am) after fasting for at least 8 hours. Plasma and peripheral blood mononuclear cell (PBMC) samples were stored at -80 °C and in liquid nitrogen tanks for each subject. The cytokine and chemokine assays on plasma samples were conducted using the MILLIPLEX® MAP Human Cytokine/Chemokine/Growth Factor Panel A MAGNETIC BEAD PANEL 96-Well Plate Assay (EMD Millipore, Billerica, MA, USA), which is based on the cutting-edge Luminex® xMAP® technology. Capture antibody-coupled magnetic beads were used to selectively bind target analytes in plasma.

The experimental plates were then wrapped in aluminum foil, gently shaken on a plate shaker, and incubated overnight at a controlled temperature of 2-8 ^°^C. After the incubation period, the plates were washed three times. Subsequently, the plates were gently agitated with assay reagents on a plate shaker and incubated for 1 hour at room temperature (24 ^°^C). Finally, the plates were assayed using Luminex® 200^TM^, HTS, and FLEXMAP 3D^®^ software (Luminex Corp., Austin, TX, USA) and carefully analyzed using a 5-parameter logistic or spline curve fitting method to ensure accurate interpretation of the results obtained from median fluorescent intensity data analysis.

**Mass Cytometry**

In summary, extracted PBMC samples were washed and stained with cisplatin-195Pt (Fluidigm, 201064) to exclude dead cells. Antibody staining was preceded by Fc receptor blocking using human TruStain FcX. All antibodies were used according to the manufacturer's recommendations. After that, cell samples were subsequently washed and incubated with cell surface antibodies at low temperatures for 30 minutes. The antibody-labeled samples were then washed and incubated in 125 nM Cell-ID Intercalator-Ir (Fluidigm, USA) and diluted in phosphate-buffered saline (PBS, Sigma-Aldrich, USA) before storage at 4 ^°^C. The samples were resuspended in double-distilled water containing EQ beads (Fluidigm, South San Francisco, USA) at a concentration of 5.5 × 10^5^ cells/ml. Finally, the pre-processed samples were analyzed by the CyTOF2 mass cytometry system (Fluidigm, South San Francisco, USA).

**Supplementary Table 1.** Seed regions definition for functional connectivity analysis.

| **Seed region number** | **Seed regions** |
| --- | --- |
| 1 | L precentral gyrus |
| 2 | R precentral gyrus |
| 3 | L superior frontal gyrus, dorsolateral |
| 4 | R superior frontal gyrus, dorsolateral |
| 5 | L superior frontal gyrus, medial |
| 6 | R superior frontal gyrus, medial |
| 7 | L superior frontal gyrus, medial orbital |
| 8 | R superior frontal gyrus, medial orbital |
| 9 | L insula |
| 10 | R insula |
| 11 | L anterior cingulate and paracingulate gyri |
| 12 | R anterior cingulate and paracingulate gyri |
| 13 | L posterior cingulate gyrus |
| 14 | R posterior cingulate gyrus |
| 15 | L hippocampus |
| 16 | R hippocampus |
| 17 | L amygdala |
| 18 | R amygdala |
| 19 | L precuneus |
| 20 | R precuneus |
| 21 | L caudate nucleus |
| 22 | R caudate nucleus |
| 23 | L superior parietal gyrus |

Abbreviations: L, left; R, right.

**Supplementary Table 2.** Enzyme-linked immunosorbent assay kits for hormones and neurotrophic factors.

| **Names** | **ELISA kits** |
| --- | --- |
| NGF | Human beta-NGF ELISA 96T |
| BDNF | Human BDNF ELISA 96T |
| GDNF | Human GDNF ELISA 96T |
| IGF-1 | Human IGF-1 ELISA 96T |
| Cortisol | Human Cortisol EIA 96T |
| CRH | Human CRH binding protein ELISA 96T |
| ACTH | Human Adrenocorticotropic Hormone EIA 96T |

ELISA, Enzyme-linked immunosorbent assay; NGF, nerve growth factor; BDNF, brain-derived neurotrophic factor; GDNF, glial cell-derived neurotrophic factor; IGF-1, insulin-like growth factor 1; CRH, corticotropin-releasing hormone; ACTH, adrenocorticotropic hormone.

**Supplementary Table 3.** Antibodies and isotopes used for mass cytometry.

| **Isotopes name** | **Antibody name** | **Isotopes name** | **Antibody name** |
| --- | --- | --- | --- |
| 209Bi | CD16 | 175Lu | PERFORIN |
| 142Ce | CD57 | 143Nd | CD45RA |
| 161Dy | KI67 | 145Nd | CD4 |
| 162Dy | FOXP3 | 146Nd | CD8 |
| 167Er | CCR7 | 141Pr | CD3 |
| 168Er | CD127 | 144Sm | CD31 |
| 170Er | HLA_DR | 148Sm | CD14 |
| 151Eu | CD107A | 149Sm | CD25 |
| 153Eu | CCR2 | 150Sm | CD86 |
| 154Gd | CD163 | 89Y | CD45 |
| 155Gd | PD1 | 172Yb | CD38 |
| 156Gd | PD_L1 |  |  |

Bi, Bismuth; Ce, Cerium; Dy, Dysprosium; Er, Erbium; Eu, Europium; Gd, Gadolinium; FOXP, Forkhead box P; CCR, C-C chemokine receptor; HLA_DR, Human Leukocyte Antigen-DR; PD, Programmed cell Death protein; Lu, Lutetium; Nd, Neodymium; Pr, Praseodymium; Sm, Samarium; Y, Yttrium; Yb, Ytterbium.

**Supplementary Table 4.** Assessment results of the symptom checklist 90 differences between the HADD and HIV control groups.

| **Symptom checklist 90** | **HADD group (N = 33)** | **HIV control group (N = 47)** | **Statistic** | ***P* value** |
| --- | --- | --- | --- | --- |
| Somatization | 1.50 (1.17 - 2.21) | 1.17 (1.00 - 1.42) | *Z* = -3.384 | 0.001^a^ |
| Obsessive compulsive | 2.20 (1.80 - 2.70) | 1.50 (1.20 - 1.90) | *Z* = -4.763 | <0.001^a^ |
| Interpersonal sensitivity | 1.89 (1.39 - 2.39) | 1.22 (1.00 - 1.67) | *Z* = -4.098 | <0.001^a^ |
| Depression | 2.31 (1.58 - 2.96) | 1.31 (1.08 - 1.69) | *Z* = -4.473 | <0.001^a^ |
| Anxiety | 1.70 (1.40 - 2.35) | 1.10 (1.00 - 1.30) | *Z* = -4.518 | <0.001^a^ |
| Hostility | 1.83 (1.33 - 2.33) | 1.17 (1.00 - 1.50) | *Z* = -4.325 | <0.001^a^ |
| Phobic anxiety | 1.29 (1.14 - 1.57) | 1.00 (1.00 - 1.29) | *Z* = -3.504 | <0.001^a^ |
| Paranoid ideation | 1.67 (1.33 - 2.09) | 1.17 (1.00 - 1.67) | *Z* = -3.221 | 0.001^a^ |
| Psychoticism | 1.80 (1.40 - 2.25) | 1.20 (1.00 - 1.40) | *Z* = -4.118 | <0.001^a^ |
| Other | 2.00 (1.36 - 2.43) | 1.29 (1.00 - 1.71) | *Z* = -3.680 | <0.001^a^ |
| Satisfied the SCL‐90 sum > 160 (yes/no) | 19/14 | 6/41 | *χ^2^* = 18.119 | <0.001^b^ |
| SCL-90 positive items > 43 (yes/no) | 22/11 | 10/37 | *χ^2^* = 16.643 | <0.001^b^ |
| At least one factor score > 2 (yes/no) | 26/7 | 10/37 | *χ^2^* = 25.909 | <0.001^b^ |
| A positive screen of SCL-90 (yes/no) | 26/7 | 11/36 | *χ^2^* = 23.922 | <0.001^b^ |

The continuous data were expressed as mean ± standard deviation or median (interquartile range) and the categorical data were expressed as number (percentage). Two-sample *t*-tests were used for continuous data with normal distribution, while Mann-Whitney *U*-tests were used for continuous data that did not obey normal distribution. Chi-square and Fisher’s exact tests were used to compare categorical variables. ^a^Mann-Whitney *U*-test; ^b^chi-square test. HADD, HIV-associated depressive disorders; HIV control, HIV-infected individuals without neuropsychiatric disorders.

**Supplementary Table 5.** The fractional amplitude of low-frequency fluctuation differences between the HADD and HIV control groups.

| **Brain region** | **Peak MNI coordinates** | | | **T value** | **Cluster Size** |
| --- | --- | --- | --- | --- | --- |
|  | **X** | **Y** | **Z** |  |  |
| **HADD > HIV control** |  |  |  |  |  |
| L superior parietal gyrus | -15 | -69 | 54 | 4.2894 | 13 |

Coordinates (X; Y; Z) refer to the peak MNI coordinates of brain regions with peak intensity. Corrected for multiple comparisons (FDR correction, voxel level *P* < 0.001, cluster level *P* < 0.05). FDR, false discovery rate; HADD, HIV-associated depressive disorders; HIV control, HIV-infected individuals without neuropsychiatric disorders; MNI, Montreal Neurological Institute; L, left; R, right.

**Supplementary Table 6.** Regional homogeneity differences between the HADD and HIV control groups.

| **Brain region** | **Peak MNI coordinates** | | | **T value** | **Cluster Size** |
| --- | --- | --- | --- | --- | --- |
|  | **X** | **Y** | **Z** |  |  |
| **HADD < HIV control** |  |  |  |  |  |
| L precentral gyrus | -36 | -27 | 63 | 4.1330 | 10 |

Coordinates (X, Y, Z) refer to the peak MNI coordinates of brain regions with peak intensity (voxel-level uncorrected *P* < 0.001). HADD, HIV-associated depressive disorders; HIV control, HIV-infected individuals without neuropsychiatric disorders; MNI, Montreal Neurological Institute; L, left; R, right.

**Supplementary Table 7.** Main effects and interaction of groups and antiviral regimens on research findings.

| **Findings** | **Group** | **INSTIs** | **Non-INSTIs** | **Main effect of groups** | | | **Main effect of antiviral regimens** | | | **Interaction of groups and antiviral regimens** | | |  |
| --- | --- | --- | --- | --- | --- | --- | --- | --- | --- | --- | --- | --- | --- |
|  |  |  |  |  |  |  |  |  |  |  |  |  |  |
|  |  |  |  |  |  |  |  |  |  |  |  |  |  |
|  |  | **mean ± SD** | **mean ± SD** | ***F*** | ***P*** | **Partial η^2^** | ***F*** | ***P*** | **Partial η^2^** | ***F*** | ***P*** | **Partial η^2^** |  |
| **fALFF** |  |  |  |  |  |  |  |  |  |  |  |  |  |
| SPG.L | HADD | 0.90 ± 0.02 | 0.91 ± 0.02 | 0.748 | 0.390 | 0.010 | 2.202 | 0.142 | 0.028 | 0.144 | 0.705 | 0.002 |  |
|  | HIV control | 0.90 ± 0.01 | 0.90 ± 0.02 |  |  |  |  |  |  |  |  |  |  |
| **ReHo** |  |  |  |  |  |  |  |  |  |  |  |  |  |
| PreCG.L | HADD | 0.79 ± 0.04 | 0.78 ± 0.04 | 4.881 | 0.030 | 0.060 | 0.041 | 0.840 | 0.001 | 2.229 | 0.140 | 0.028 |  |
|  | HIV control | 0.80 ± 0.04 | 0.81 ± 0.05 |  |  |  |  |  |  |  |  |  |  |
| **FC** |  |  |  |  |  |  |  |  |  |  |  |  |  |
| PreCG.R-CUN.R | HADD | 0.21 ± 0.11 | 0.20 ± 0.12 | 1.732 | 0.192 | 0.022 | 0.048 | 0.826 | 0.001 | 0.014 | 0.905 | <0.001 |  |
|  | HIV control | 0.25 ± 0.15 | 0.25 ± 0.15 |  |  |  |  |  |  |  |  |  |  |
| PreCG.R-MOG.L | HADD | 0.21 ± 0.10 | 0.20 ± 0.18 | 5.866 | 0.018 | 0.072 | 0.001 | 0.969 | <0.001 | 0.058 | 0.811 | 0.001 |  |
|  | HIV control | 0.28 ± 0.14 | 0.29 ± 0.15 |  |  |  |  |  |  |  |  |  |  |
| PreCG.R-MFG.L | HADD | 0.14 ± 0.08 | 0.15 ± 0.07 | 0.096 | 0.758 | 0.001 | 0.306 | 0.582 | 0.004 | 0.000 | 0.988 | <0.001 |  |
|  | HIV control | 0.15 ± 0.09 | 0.16 ± 0.12 |  |  |  |  |  |  |  |  |  |  |
| PreCG.R-IPL.L | HADD | 0.19 ± 0.12 | 0.16 ± 0.10 | 1.274 | 0.263 | 0.016 | 0.476 | 0.492 | 0.006 | 0.057 | 0.811 | 0.001 |  |
|  | HIV control | 0.21 ± 0.13 | 0.20 ± 0.15 |  |  |  |  |  |  |  |  |  |  |
| ORBsupmed.R-IPL.L | HADD | 0.01 ± 0.09 | 0.05 ± 0.08 | 3.512 | 0.065 | 0.044 | 1.380 | 0.244 | 0.018 | 0.393 | 0.533 | 0.005 |  |
|  | HIV control | 0.07 ± 0.11 | 0.08 ± 0.12 |  |  |  |  |  |  |  |  |  |  |
| INS.R-CAL.R | HADD | 0.04 ± 0.12 | 0.08 ± 0.12 | 1.705 | 0.196 | 0.022 | 1.703 | 0.196 | 0.022 | 0.003 | 0.957 | <0.001 |  |
|  | HIV control | 0.08 ± 0.15 | 0.13 ± 0.12 |  |  |  |  |  |  |  |  |  |  |
| INS.R-LING.R | HADD | 0.04 ± 0.11 | 0.08 ± 0.11 | 3.047 | 0.085 | 0.039 | 1.181 | 0.281 | 0.015 | 0.013 | 0.909 | <0.001 |  |
|  | HIV control | 0.10 ± 0.14 | 0.13 ± 0.14 |  |  |  |  |  |  |  |  |  |  |
| INS.R-PoCG.R | HADD | 0.05 ± 0.08 | 0.08 ± 0.08 | 1.734 | 0.192 | 0.022 | 0.546 | 0.462 | 0.007 | 0.198 | 0.657 | 0.003 |  |
|  | HIV control | 0.09 ± 0.12 | 0.10 ± 0.12 |  |  |  |  |  |  |  |  |  |  |
| PCG.R-ITG.L | HADD | 0.06 ± 0.09 | 0.09 ± 0.05 | 0.028 | 0.866 | <0.001 | 1.335 | 0.252 | 0.017 | <0.001 | 0.992 | <0.001 |  |
|  | HIV control | 0.06 ± 0.08 | 0.08 ± 0.12 |  |  |  |  |  |  |  |  |  |  |
| **Cytokine** |  |  |  |  |  |  |  |  |  |  |  |  |  |
| IFN-gamma | HADD | 2.15 ± 1.85 | 28.98 ± 98.79 | 4.782 | 0.032 | 0.060 | 1.549 | 0.217 | 0.020 | 0.468 | 0.496 | 0.006 |  |
|  | HIV control | 1.31 ± 0.92 | 1.14 ± 0.39 |  |  |  |  |  |  |  |  |  |  |
| **Immune** |  |  |  |  |  |  |  |  |  |  |  |  |  |
| cluster14 | HADD | 20.78 ± 15.48 | 15.17 ± 17.05 | 4.481 | 0.038 | 0.056 | 1.318 | 0.254 | 0.017 | 0.286 | 0.594 | 0.004 |  |
|  | HIV control | 11.94 ± 12.56 | 9.89 ± 10.22 |  |  |  |  |  |  |  |  |  |  |
| cluster1_CD38 | HADD | 3.24 ± 0.50 | 3.28 ± 0.41 | 6.160 | 0.015 | 0.075 | 0.130 | 0.719 | 0.002 | 0.006 | 0.936 | <0.001 |  |
|  | HIV control | 2.91 ± 0.55 | 2.97 ± 0.60 |  |  |  |  |  |  |  |  |  |  |
| cluster3_perforin | HADD | 2.49 ± 0.40 | 2.62 ± 0.60 | 2.575 | 0.113 | 0.033 | 3.259 | 0.075 | 0.041 | 0.477 | 0.492 | 0.006 |  |
|  | HIV control | 2.23 ± 0.51 | 2.52 ± 0.40 |  |  |  |  |  |  |  |  |  |  |
| cluster3_CD38 | HADD | 0.31 ± 0.56 | 0.42 ± 0.48 | 4.846 | 0.031 | 0.060 | 0.648 | 0.423 | 0.008 | 0.125 | 0.725 | 0.002 |  |
|  | HIV control | 0.14 ± 0.25 | 0.18 ± 0.28 |  |  |  |  |  |  |  |  |  |  |
| cluster4_CD38 | HADD | 0.41 ± 0.32 | 0.47 ± 0.34 | 5.116 | 0.027 | 0.063 | 0.096 | 0.758 | 0.001 | 0.321 | 0.573 | 0.004 |  |
|  | HIV control | 0.29 ± 0.24 | 0.27 ± 0.28 |  |  |  |  |  |  |  |  |  |  |
| cluster5_perforin | HADD | 3.27 ± 0.46 | 3.43 ± 0.42 | 4.632 | 0.035 | 0.057 | 1.337 | 0.251 | 0.017 | 0.069 | 0.794 | 0.001 |  |
|  | HIV control | 3.06 ± 0.51 | 3.16 ± 0.35 |  |  |  |  |  |  |  |  |  |  |
| cluster12_perforin | HADD | 0.82 ± 0.66 | 0.86 ± 0.78 | 2.117 | 0.150 | 0.027 | 2.669 | 0.106 | 0.034 | 1.931 | 0.169 | 0.025 |  |
|  | HIV control | 0.41 ± 0.43 | 0.85 ± 0.68 |  |  |  |  |  |  |  |  |  |  |
| cluster14_CD38 | HADD | 1.34 ± 0.20 | 1.35 ± 0.17 | 5.771 | 0.019 | 0.071 | 0.283 | 0.597 | 0.004 | 0.091 | 0.763 | 0.001 |  |
|  | HIV control | 1.21 ± 0.19 | 1.25 ± 0.19 |  |  |  |  |  |  |  |  |  |  |
| cluster19_CD38 | HADD | 3.15 ± 0.72 | 3.23 ± 0.40 | 3.635 | 0.060 | 0.046 | 2.263 | 0.137 | 0.029 | 0.978 | 0.326 | 0.013 |  |
|  | HIV control | 2.74 ± 0.62 | 3.09 ± 0.42 |  |  |  |  |  |  |  |  |  |  |

Continuous data were presented as mean ± SD. Abbreviations: SD, standard deviation; HADD, HIV-associated depressive disorders; HIV control, HIV-infected individuals without neuropsychiatric disorders; ALFF, fractional amplitude of low-frequency fluctuation; ReHo, regional homogeneity; FC, functional connectivity; SPG.L, left superior parietal gyrus; PreCG.L, left precentral gyrus; PreCG.R, right precentral gyrus; CUN.R, right cuneus; MOG.L, left middle occipital gyrus; MFG.L, left middle frontal gyrus; IPL.L, left inferior parietal, but supramarginal and angular gyri; ORBsupmed.R, right medial orbital part of the superior frontal gyrus; INS.R, right insula; CAL.R, right calcarine fissure and surrounding cortex; LING.R, right lingual gyrus; PoCG.R, right postcentral gyrus; PCG.R, right posterior cingulate gyrus; ITG.L, left inferior temporal gyrus; IFN, interferon; Cluster 14 represented nonclassical monocytes, cluster 1 represented nonclassical monocytes, cluster 3 represented CD8^+^ effector memory T cells, cluster 4 represented CD8^+^ naïve T cells, cluster 5 represented nonclassical monocytes, cluster 12 represented double negative T cells, and cluster 19 represented nonclassical monocytes.
